# HIV-1 Specific IgA Detected in Vaginal Secretions of HIV Uninfected Women Participating in a Microbicide Trial in Southern Africa Are Primarily Directed Toward gp120 and gp140 Specificities

**DOI:** 10.1371/journal.pone.0101863

**Published:** 2014-07-23

**Authors:** Kelly E. Seaton, Lamar Ballweber, Audrey Lan, Michele Donathan, Sean Hughes, Lucia Vojtech, M. Anthony Moody, Hua-Xin Liao, Barton F. Haynes, Christine G. Galloway, Barbra A. Richardson, Salim Abdool Karim, Charlene S. Dezzutti, M. Juliana McElrath, Georgia D. Tomaras, Florian Hladik

**Affiliations:** 1 Duke Human Vaccine Institute, Durham, North Carolina, United States of America; 2 Department of Obstetrics and Gynecology, University of Washington, Seattle, Washington, United States of America; 3 Vaccine and Infectious Disease Division, Fred Hutchinson Cancer Research Center, Seattle, Washington, United States of America; 4 Department of Biostatistics, University of Washington, Seattle, Washington, United States of America; 5 CAPRISA - Centre for the AIDS Programme of Research in South Africa, Nelson R Mandela School of Medicine, University of KwaZulu-Natal, Durban, South Africa; 6 Department of Epidemiology, Columbia University, New York, New York, United States of America; 7 Department of Obstetrics, Gynecology and Reproductive Sciences, University of Pittsburgh School of Medicine, Pittsburgh, Pennsylvania, United States of America; 8 Department of Medicine, University of Washington, Seattle, Washington, United States of America; 9 Department of Laboratory Medicine, University of Washington, Seattle, Washington, United States of America; 10 Department of Global Health, University of Washington, Seattle, Washington, United States of America; Karolinska Institutet, Sweden

## Abstract

**Background:**

Many participants in microbicide trials remain uninfected despite ongoing exposure to HIV-1. Determining the emergence and nature of mucosal HIV-specific immune responses in such women is important, since these responses may contribute to protection and could provide insight for the rational design of HIV-1 vaccines.

**Methods and Findings:**

We first conducted a pilot study to compare three sampling devices (Dacron swabs, flocked nylon swabs and Merocel sponges) for detection of HIV-1-specific IgG and IgA antibodies in vaginal secretions. IgG antibodies from HIV-1-positive women reacted broadly across the full panel of eight HIV-1 envelope (Env) antigens tested, whereas IgA antibodies only reacted to the gp41 subunit. No Env-reactive antibodies were detected in the HIV-negative women. The three sampling devices yielded equal HIV-1-specific antibody titers, as well as total IgG and IgA concentrations. We then tested vaginal Dacron swabs archived from 57 HIV seronegative women who participated in a microbicide efficacy trial in Southern Africa (HPTN 035). We detected vaginal IgA antibodies directed at HIV-1 Env gp120/gp140 in six of these women, and at gp41 in another three women, but did not detect Env-specific IgG antibodies in any women.

**Conclusion:**

Vaginal secretions of HIV-1 infected women contained IgG reactivity to a broad range of Env antigens and IgA reactivity to gp41. In contrast, Env-binding antibodies in the vaginal secretions of HIV-1 uninfected women participating in the microbicide trial were restricted to the IgA subtype and were mostly directed at HIV-1 gp120/gp140.

## Introduction

Only approximately one in twenty to one in two thousand unprotected sexual encounters with an HIV-1-infected partner leads to systemic HIV-1 infection in the recipient [1], [2]. There is evidence, though, that exposures to HIV-1 that fail to establish infection can still exert immunological effects. For example, HIV-1-specific T cells have been detected in highly HIV-1-exposed individuals who remain seronegative [3]–[13]. Some studies have also found mucosal HIV-1-specific neutralizing IgA in the cervicovaginal lavage and seminal fluid of HIV-1-negative individuals with HIV-1-infected partners [11], [14]–[17]. Such immunological responses may serve as markers of prior HIV-1 exposure. Risk status (i.e. level of HIV-1 exposure) is an important consideration when recruiting subjects for efficacy trials of HIV-1 prevention strategies – the lower the population risk of HIV-1 infection, the larger the study cohorts must be to conclusively test effectiveness. The presence of HIV-1-specific immunity at a site of potential HIV-1 transmission might also confer some protection from infection [18]. Understanding such immune responses could be of value for HIV-1 vaccine design.

HIV-1-specific antibody titers are in general easier to measure than HIV-1-specific T cell function, but there is little clear guidance regarding the optimal specimen types and sampling devices for the assessment of mucosal antibodies in HIV-1 vaccine and microbicide clinical trials. Cervicovaginal lavage (CVL) has been used for the quantification of proteins in genital secretions. However, CVL sampling is more complicated than swabbing, and the dilution of mucosal secretions and the resulting variability in recovered sample volumes can decrease the sensitivity of protein detection and pose a challenge for data interpretation [19]–[21].

Therefore in this study, we compared three direct vaginal sampling devices not requiring a lavage: Dacron swabs, flocked nylon swabs and Merocel sponges. Using a cohort of five HIV-positive and five HIV-negative women in Seattle, we determined total IgG and IgA concentrations obtained from each device, and then compared antibody reactivity to a panel of eight HIV-1 Env antigens with an HIV-1-specific binding antibody multiplex assay, previously utilized for both serum/plasma and mucosal specimens [22]–[25]. We then examined HIV-1-specific antibody responses in swabs obtained from African women participating in the control arm of the HPTN 035 microbicide trial, a Phase IIb study designed to assess the effectiveness of 0.5% PRO2000 gel and BufferGel for the prevention of HIV-1 infection [26]. We found HIV-1-specific IgA, but not IgG, antibodies in the vaginal secretions of nine of 57 HPTN 035 participants. These mucosal HIV-1-specific IgA antibodies could contribute to protection in these women or be a marker of another protective function; thus, defining mucosal HIV-specific antibodies in future microbicide trials should be of major interest to the HIV prevention field.

## Methods

### Clinical Cohorts and Ethical Approval

Two cohorts of women were enrolled in this study following review and all protocols were approved by the Fred Hutchinson Cancer Research Center Institutional Review Board, Duke University Medical Center IRB, and local IRBs at each of the HPTN 035 sites. All participants gave written informed consent to be a part of the study. The Seattle cohort included five HIV-uninfected women at low risk for HIV-1 infection and five women with HIV-1 clade B infection who were on antiretroviral treatment (average blood CD4 count 837/µl). The African cohort comprised a total of 58 participants in the control, condom-use-only, arm of the microbicide trial HPTN 035 (ClinicalTrials.gov registration ID NCT00074425). Vaginal Dacron swabs from approximately 10 women from six HPTN 035 sites were included for analysis: Blantyre and Lilongwe, Malawi; Durban and Hlabisa, South Africa; and Harare and Chitungwiza, Zimbabwe. One of the 58 women was found to be clade C HIV-1 seropositive during the trial and is included here as a positive control. The demographics of the HPTN 035 study participants, the study protocol and the results of the clinical study are described elsewhere [26], [27]. Vaginal smears were Gram stained for bacterial vaginosis (BV) diagnosis by Nugent scoring. Gram stains with a Nugent score of 4–6 or ≥7 were classified as signifying abnormal flora or BV, respectively.

### Swab collection and processing in the Seattle cohort

In the Seattle cohort, three types of swabs were used for the collection of vaginal secretions: Dacron polyester-tipped swabs (Fisher Scientific Cat. No. 23-400-122), flocked nylon swabs (Copan Diagnostics; Fisher Scientific Cat. No. 23-600-957) and Merocel sponges (Merocel Eye Spears, Beaver-Visitec International, Item No. 400101). Six swabs were taken from each of five HIV-1-infected and five HIV-uninfected women during two sessions that were spaced one hour apart. Three swabs, one of each type, were collected in each session. Half of the women were randomly assigned to begin session 1 with Dacron swabs followed by flocked swabs; the other half began session 1 with flocked swabs followed by Dacron swabs. This swab order was reversed in session 2. In both sessions, Merocel sponges were the third and last sample type obtained. Swabs were placed into the posterior vaginal fornix until visibly saturated, but never longer than 1 minute. Swabs were immediately placed into 500 µl of elution buffer (cold phosphate-buffered saline (PBS) containing 1% protease inhibitor cocktail (Calbiochem Set I), 10% Igepal (Sigma) and 0.25% bovine serum albumin (Sigma)). After transport on ice to the laboratory, secretions were eluted on the same day. Swabs or Merocel sponges were transferred into a pre-chilled 0.45 µm SPIN-X filter (Corning Life Sciences). Swab or sponge handles were clipped off, the transport buffer was transferred to the SPIN-X filters, and the SPIN-X filters were spun at 13,000×g for 15 min at 4°C. 300 µl of elution buffer were added, the SPIN-X filters were incubated on ice for 5 min, and spun at 13,000×g for 30 min at 4°C. Filter and sponges were removed and discarded. Eluates were distributed at 100 µl per vial and stored at −80°C until assayed.

### Swab collection and processing in HPTN 035

In HPTN 035, vaginal Dacron swabs were collected as described previously [27]. In brief, during a quarterly pelvic exam, a Dacron swab was applied to the posterior fornix of the vagina to saturate the tip with fluid and then placed in a cryovial containing 400 µl of PBS. The cryovials were stored at −80°C at the sites and shipped to the Microbicide Trials Network (MTN) Network Laboratory after the primary study results were available [26]. Swabs were thawed on wet ice, vortexed, and compressed against the side of the tube to elute secretions. Swabs were removed and placed upside down in a new tube and centrifuged at 700×g for 10 min at 4°C to elute remaining secretions. Eluates of both tubes were combined, using 200 µl of PBS for rinsing. The combined eluate was centrifuged again to pellet particles. Eluates (approximately 350 µl total) were stored in aliquots at −80°C until assayed.

### Multiplex assay for detection of HIV-1-binding antibodies

The HIV-1 binding antibody multiplex assay has been previously described [23]–[25], [28]–[30]. Briefly, HIV-1 antigens were conjugated to polystyrene beads (Bio-Rad) and binding of IgG or IgA in the eluates to the bead-conjugated HIV-1 antigens was detected by mouse anti-human IgG-PE (Southern Biotech) or goat anti-human IgA-PE (Jackson Immunoresearch). To measure HIV-1-specific IgA, eluates were first depleted of IgG using Protein G high-performance MultiTrap plates (General Electric) according to the manufacturer’s instructions. After incubation of beads and eluates, the beads were washed and acquired on a Bio-Plex 200 instrument (Bio-Rad). HIV-1-specific binding antibodies for each antigen were measured as Mean Fluorescence Intensity (MFI). Total IgG and IgA antibody measurements for calculating specific activity were performed using a Bio-Plex Pro Human Isotyping 7-plex panel (Bio-Rad) according to the manufacturer’s instructions.

Positivity calls per HIV-1 antigen per antibody isotype were based on preset criteria determined from HIV-1 negative samples in the Seattle cohort and were consistent with historical positivity criteria for mucosal responses. An HIV-1-specific antibody response was considered positive if it met the positivity criteria for both MFI and specific activity. Positive MFI responses were defined as greater than the mean MFI plus 5 standard deviations of the antigen with the highest background among the 30 samples taken from the five uninfected women in Seattle. To account for subject-to-subject variation in fluid collected (including levels affected by timing of the menstrual cycle and contraceptive use), MFI values were normalized to total IgG or IgA and computed as specific activity (antigen specific MFI/ng ml^−1^ total IgG or IgA). All total antibody measurements were assesed in samples diluted 1∶3 in assay diluent (Bio-Rad). Positive specific activity responses were defined as greater than the mean plus five standard deviations of the specific activities measured in the five seronegative individuals in the Seattle cohort.

### Reagents for antibody measurements

Antibody responses were determined for the following antigens: Clade B gp41 Immunodominant epitope tetramer (gp41 ID epitope) (Biotin -CRVLAVERYLRDQQLLGIWGCSGKLICTTAVPWNASWSNKSLNKI), 2F5 mAb epitope tetramer (Biotin-GGGQQEKNEQELLELDKWASLWN), clade B MPER tetramer (MPR.03) (KKKNEQELLELDKWASLWNWFDITNWLWYIRKKK-Biotin) (containing the linear binding sites for MPER neutralizing antibodies), and clade C MPER (MPR.C956) tetramer (Biotin-NEQELLELDKWASLWNWFNITNWLW) (derived from a clade C sequence and containing the linear binding sites MPER neutralizing antibodies (tetramers provided by Dr. Anthony Moody, Duke Human Vaccine Institute; [31],[32]); Consensus gp120 Env (Con6 gp120), clade C gp140 Env trimer (1086 Trimer) (a transmitted/founder sequence envelope sequence from a clade C infected patient from Malawi, [33] and a Group M consensus gp140 Env (ConS gp140) [34]–[36] (envelope proteins provided by Drs. Barton Haynes and Larry Liao, Duke Human Vaccine Institute). The consensus sequence for Group M is central to all circulating clades, and reacts well with sera from all subtypes [34]–[36] and was found to be similar to autologous envelopes in detection of the initial antibody response in acute infections [23].

Clade B HIV-1_MN_ recombinant gp41 protein was commercially available (Immunodiagnostics). Positive controls included titration of purified pooled HIV-1^+^ immunoglobulin (HIVIG), 2F5 monoclonal antibody (mAb) IgA, 2F5 mAb IgG, 4E10 IgG, b12 IgG (all from Polymun Scientific), b12 IgA (kindly provided by Drs. Ann Hessell and Dennis Burton, The Scripps Research Institute), and 7B2 mAb IgG (Dr. James Robinson, Tulane University). Negative controls included matched seronegative samples, bare tetramers, blank beads, and normal human serum (NHS) (Sigma).

### Statistical analysis

For the Seattle pilot cohort, ANOVA with fixed effects was applied to assess the influence of swab type, time point of sample collection and subject HIV serostatus on log_10_ total IgG and IgA concentrations. ANOVA with fixed effects was also used to assess the influence of swab type and time point of sample collection on log_10_ HIV-1-specific IgG activities. For the HPTN 035 cohort, the effect of study country or vaginal flora Nugent scoring on the presence of vaginal HIV-1-reactive IgA antibodies was tested by Chi-square test or Fisher’s exact test, respectively.

## Results

### Comparison of three sampling devices for measuring total and HIV-1 envelope-specific IgG and IgA antibodies in vaginal secretions

**Figure 1:**
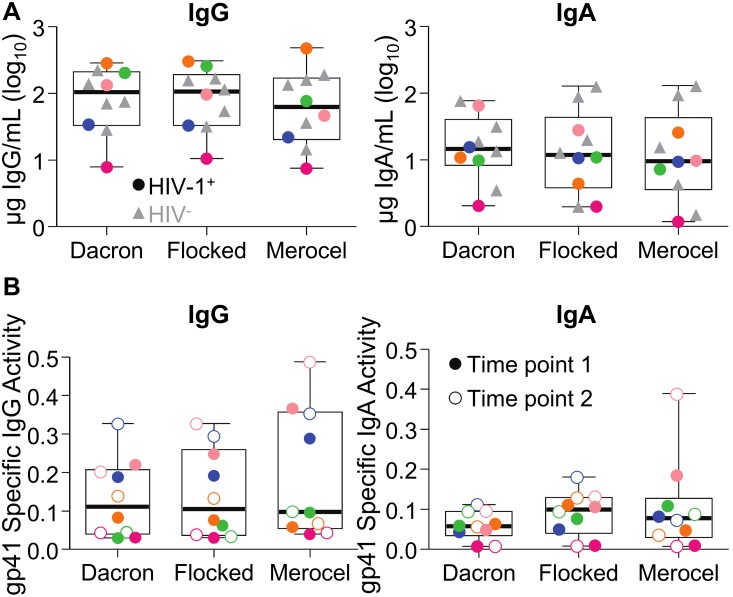
Total and HIV-1-specific IgG and IgA in vaginal secretions do not differ by sampling device or timepoint in the same women. (A) Dacron swabs, flocked swabs, and Merocel sponges were collected from 5 HIV-1 infected (colored circles) and 5 HIV uninfected (grey triangles) women and the eluate was analyzed for total IgG (left) and total IgA (right). Symbols represent the average of two collection time points spaced one hour apart, for each woman and sampling device. For HIV-1 infected women, colors indicate specific individuals. (B) Samples from the first time point (filled circles) and one hour later (open circles) from the 5 HIV-1 infected women were analyzed for gp41-specific IgG (left) and IgA (right) activity, calculated as antigen-specific mean fluorescence intensity (MFI)/ng ml^−1^ total IgG or IgA. Colors indicate specific individuals. The box-and-whisker plots represent median, 25^th^ and 75^th^ percentiles, and range.

Dacron polyester-tipped swabs, flocked nylon swabs and Merocel sponges were obtained from 5 HIV-1 infected women and 5 HIV uninfected women (Seattle cohort). Six samples were taken from each woman, one of each type in a first session, and one of each type again one hour later. The amount of total IgG and IgA collected from each sampling device did not differ by device type (p = 0.7 for IgG; p = 0.8 for IgA) or time point of sampling (ANOVA with fixed effects, p = 0.6 for IgG; p = 0.4 for IgA) (**Figure 1A**). The total amount of IgG also did not differ by HIV serostatus (p = 0.5), however the total amount of IgA was significantly higher in the HIV-negative subjects (mean log_10_ 4.24 ng/ml±0.65 versus log_10_ 3.91±0.48; p = 0.03) (**Figure 1A**). In these samples, we additionally examined HIV-1-specific activity against a panel of eight Env antigens covering multiple epitopes in gp41, the gp41 membrane proximal external region (MPER), gp120 and gp140. HIV-1-specific IgG activity for each antigen did not differ by device type or time point of sampling (all F-test p values>0.05). There were too few positive values to statistically evaluate differences in HIV-1-specific IgA activity. Specific activities for gp41-binding IgG (**Figure 1B**, left panel) and IgA (**Figure 1B**, right panel) are shown as a representative antigen. In conclusion, Dacron swabs, flocked swabs and Merocel sponges appear equally suitable for measuring total immunoglobulins and HIV-1-specific IgG and IgA in vaginal secretions.

### Comparison of HIV-1 envelope-specific IgG to IgA responses in the vagina of HIV-1 infected women

**Figure 2:**
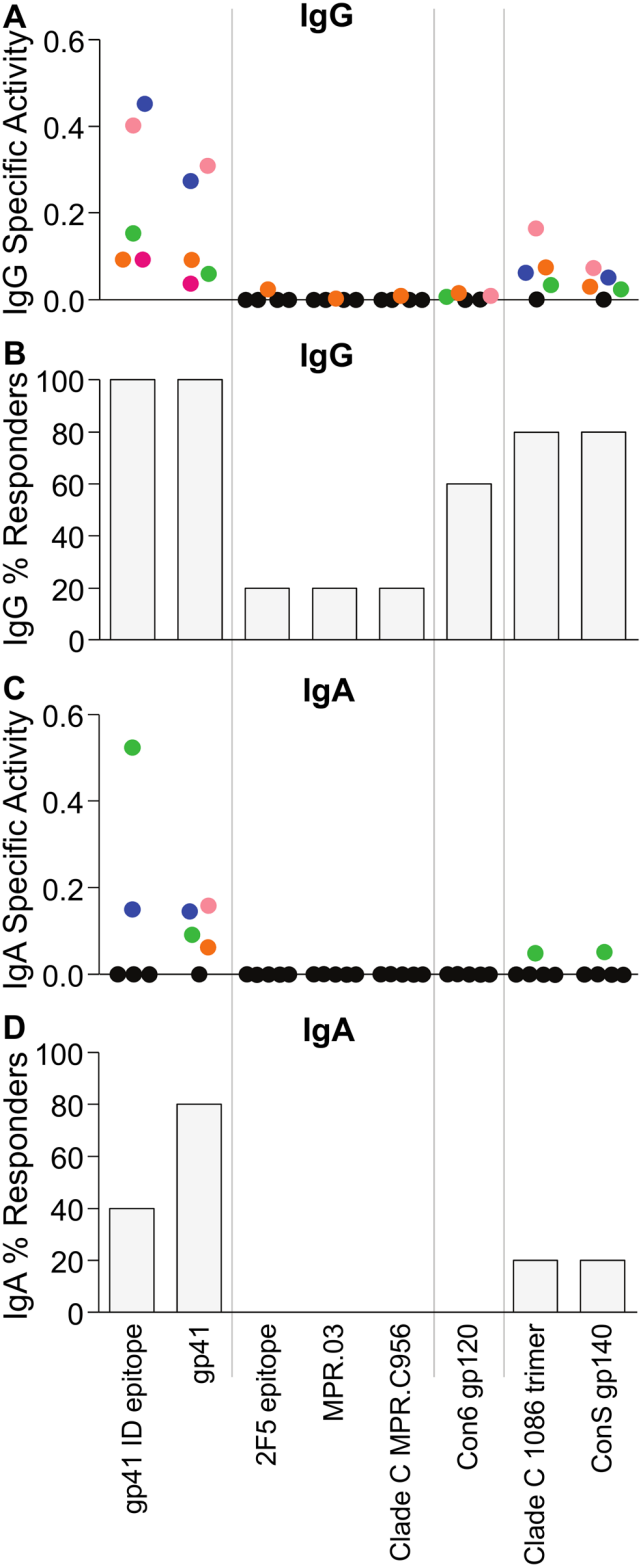
HIV-1 infection induces a broader range of HIV-1 Env-specific IgG than IgA antibodies in vaginal secretions. Vaginal secretions were collected from 5 HIV-1 infected women and analyzed for IgG (A–B) and IgA (C–D) binding to the indicated HIV-1 Env antigens. Antigens not specified as Clade C or consensus (Con6 and ConS) are derived from Clade B viruses. (A) Average HIV-1 antigen-specific IgG activity from the combination of the three sampling devices and two time points, calculated as antigen-specific MFI/ng ml^−1^ total IgG. Black symbols represent negative responses and colored symbols indicate positive responses, with the colors corresponding to specific individuals. Samples that did not meet the positivity criteria are displayed at zero for specific activity. (B) Percentage of women with positive IgG binding. (C) Average HIV-1 antigen-specific IgA activity, calculated as antigen-specific MFI/ng ml^−1^ total IgA. (D) Percentage of women with positive IgA binding.

HIV-1 infection induced a broad range of envelope-specific IgG responses in the vaginal mucosa of the five HIV-1 infected women in Seattle (**Figure 2A and B**). These responses favored gp41 and the gp41 ID epitope (5/5 women tested positive); however significant responses were also seen against the gp120 antigen Con6 gp120 (3/5), and the gp140 antigens Clade C 1086 trimer (4/5) and ConS gp140 (4/5). Only one woman had IgG antibodies against the three membrane proximal external region (MPER) antigens. IgA responses were seen at a lower frequency (**Figure 2C and D**). HIV-1-specific IgA activity was predominantly directed toward gp41 (4/5 women) and the gp41 ID epitope (2/5). One woman had gp140-specific IgA against the Clade C 1086 trimer and ConS gp140 antigens. None of the women had vaginal IgA antibodies specific to the three MPER antigens or to gp120. None of the 30 specimens taken from the five HIV uninfected women had IgG or IgA antibodies to any of the tested HIV-1 antigens (not shown). These data indicate that the HIV-1 binding antibody multiplex assay detects vaginal HIV-1 specific antibodies in a highly specific manner, and that the vaginal IgA response to HIV-1 infection is markedly narrower than the vaginal IgG response.

### HIV-1 envelope-specific binding antibody responses in the vaginal mucosa of African women participating in the HPTN 035 microbicide trial

**Figure 3:**
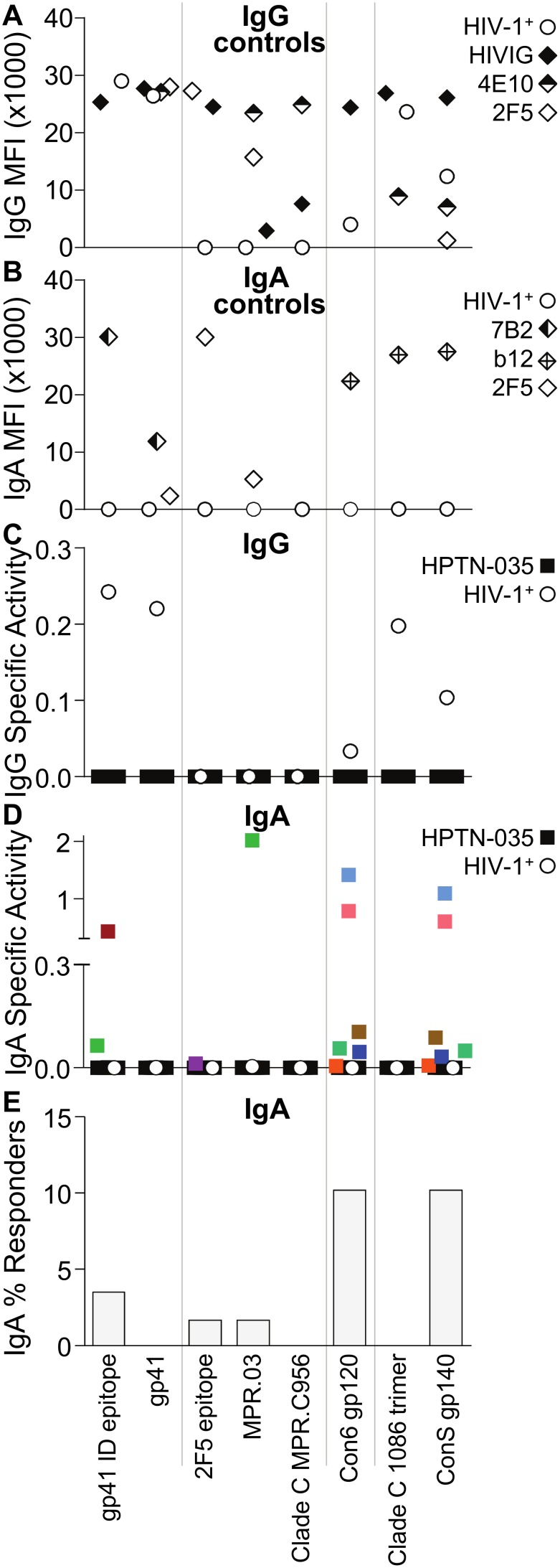
HIV-1 exposure elicits anti-Env IgA but not IgG antibodies in the vaginal mucosa. Vaginal Dacron swabs were collected from 57 HIV seronegative women enrolled in the HPTN 035 microbicide trial and analyzed for IgG (C) and IgA (D–E) binding to the indicated HIV-1 Env antigens. (A) Positive IgG controls. Binding of purified pooled HIV-1^+^ immunoglobulin (HIVIG), 4E10 monoclonal IgG (50 µg/ml), 2F5 monoclonal IgG (16 µg/ml) and swabs from an HIV-1 infected HPTN 035 participant (HIV^+^) in mean fluorescent intensity (MFI). (B) Positive IgA controls. Binding of 7B2 monoclonal IgA (1 µg/ml), b12 monoclonal IgA (20 µg/ml) and 2F5 monoclonal IgA (1 µg/ml) in MFI. Lack of IgA binding from an HIV-1 infected HPTN 035 participant (HIV^+^) is also shown. (C) HIV-1 antigen-specific IgG activity, calculated as antigen-specific MFI/ng ml^−1^ total IgG, in 57 women participating in HPTN 035. One additional HIV-1 infected HPTN 035 participant (HIV^+^) is also shown. (D) HIV-1 antigen-specific IgA activity, calculated as antigen-specific MFI/ng ml^1^ total IgA. For the 57 HPTN 035 women (squares), black symbols signify negative responses and colored symbols signify positive responses, with colors indicating specific women. Samples that did not meet the positivity criteria are displayed at zero for specific activity. One additional HIV-1 infected HPTN 035 participant (HIV^+^) is also shown (open circles). (E) Percentage of the 57 HPTN 035 women with positive IgA binding.

Having demonstrated the specificity of our assay to detect HIV-1-reactive antibodies in vaginal secretions, as well as the suitability of Dacron swabs to obtain secretions for performing these measurements, we next examined vaginal HIV-1-specific antibody responses in a cohort of African women enrolled in the HPTN 035 Phase IIb clinical trial. Of the 58 women we analyzed from this cohort, 57 women remained uninfected while one woman seroconverted. Vaginal secretions from the sole seroconverter contained HIV-1-specific IgG (**Figure 3A**) but not IgA antibodies (**Figure 3B**). Pooled HIV^+^ immunoglobulin, as well as the control IgG antibodies 4E10 and 2F5, showed binding to their respective antigens (**Figure 3A**). Likewise, the control IgA antibodies 7B2, b12 and 2F5 bound their respective antigens (**Figure 3B**).

None of the 57 HIV uninfected women had HIV-1 envelope-reactive vaginal IgG antibodies (**Figure 3C**). Nine women (15.8%) had HIV-1 envelope-reactive IgA antibodies in their vaginal secretions (**Figure 3D and E**). IgA antibodies to both Con6 gp120 and ConS gp140 were detected in six women (10.5%), and IgA antibodies from two additional women were specific for the gp41 ID epitope (3.5%). Secretions from one of these two women also reacted to the MPR.03 epitope (1.8%). Vaginal secretions from the ninth woman reacted to the 2F5 epitope (1.8%). These data indicate the presence of HIV-1 envelope-reactive IgA but not IgG antibodies in vaginal secretions of ∼16% of African women participating in the control arm of the HPTN 035 microbicide trial. While antibodies from each woman reacted to only one or two HIV-1 antigens, collectively, antibodies from the nine IgA-positive women reacted to five of the eight HIV-1 envelope antigens tested.

### Relationship of vaginal HIV-1-reactive IgA antibodies in HIV-negative women to HPTN 035 study site and country and to bacterial vaginosis

Particularly high HIV-1 incidence rates have been reported for South Africa, with a reported prevalence of 21.1% in young women aged 20–24 (2008), and a prevalence up to 39.5% in pregnant women in KwaZulu Natal (2010) [37], [38]. Consequently, the likely higher HIV-1 exposure rates in South Africa could lead to the emergence of vaginal HIV-1-reactive IgA antibodies more often than in countries with lower HIV-1 incidence. We thus investigated the geographic distribution of the nine women with vaginal HIV-1-reactive IgA antibodies were seen. In South Africa, 3/9 women in Durban (33.3%) and 1/9 women in Hlabisa (11.1%); in Zimbabwe, 3/10 women in Chitungwiza (30%) and 0/10 women in Harare (0%); and in Malawi, 1/9 women in Lilongwe (11.1%) and 1/10 women in Blantyre (10%), were IgA positive. Thus, 22.2% of South African, 15% of Zimbabwean and 10.5% of Malawian women tested positive for vaginal HIV-1-reactive IgA antibodies, with no significant differences between countries (Chi square p = 0.62).

We also investigated whether a relationship between vaginal HIV-1-reactive IgA status and bacterial vaginosis (BV) existed. Twenty of the 57 HIV-1 uninfected HPTN 035 women had abnormal vaginal flora (Nugent score 4–6) and seven had BV (Nugent score≥7). Among the nine women with HIV-1-reactive IgA antibodies, two had abnormal flora and one had BV, while 18 of the other 48 women had abnormal flora (Fisher’s exact p = 0.47) and six had BV (p = 1.0). Thus, abnormal vaginal flora and bacterial vaginosis did not correlate with the presence of vaginal HIV-1 envelope-reactive IgA antibodies.

## Discussion

Our study confirms a number of prior reports of HIV-1-specific IgA in the genital tracts of HIV-1-exposed seronegative women [6], [14], [17], [39]–[42]. Between 38% and 82% of highly exposed seronegative (HESN) women [6], [41], [42] have been reported to have genital HIV-1-specific IgA, although one study of uninfected sex workers from the Gambia failed to detect any vaginal antibody responses against HIV-1 [43]. In our study, 9/57 (15.8%) women had vaginal HIV-1-specific IgA antibodies. Reasons for divergent frequencies in different studies and cohorts have been discussed elsewhere [16], but actual exposure rates to HIV-1 should be a major determinant. It is not surprising that IgA frequencies in our cohort were at the lower end of the reported range, because HPTN 035 study subjects [26], including the 57 women we studied, were not screened for self-reported risk factors such as frequent unprotected sex, a known HIV-infected partner and/or commercial sex work. Thus, HPTN 035 study women did not fit the HESN definition [44], [45] and their risk profile was on average likely lower than that of HESN individuals. Moreover, sample collection and storage methods, in addition to the conservative, two-tiered definition of antibody positivity, may have contributed to the relatively low frequency of HPTN 035 women with positive HIV-1-specific vaginal IgA. Additionally the hormonal cycle can influence both IgG and IgA antibody levels [46], [47]. One limitation of our study with HPTN 035 samples was that due to study size and inclusion criteria (∼3099 female participants with some contraceptive use) the sample collections were not synchronized around the participant’s menstrual cycle. We controlled for antibody levels among participants in our analyses by factoring in total antibody levels along with the HIV-1 specific antibody calculations.

Nevertheless, vaginal swabs from ∼16% of the uninfected HPTN 035 women we tested were positive for HIV-1-specific IgA antibodies, whereas none of the 57 women were positive for HIV-1-specific IgG antibodies. This indicates that the presence of HIV-1-specific mucosal IgA could be used as an indicator of HIV-1 exposure in subjects participating in HIV prevention trials [48]. Additionally, our study demonstrated that vaginal swabs routinely obtained during a microbicide trial and stored frozen for several years can be used for that purpose. While this was the first time that HIV-1-specific mucosal antibodies were evaluated in a microbicide trial, similar studies should be pursued in ongoing and future trials of topical pre-exposure prophylaxis (PrEP). Of particular interest will be the question of whether efficacious topical PrEP, which as of yet has not been developed, might contribute to a mucosal state of HIV-1-specific immunity by exposures to microbicide-inactivated HIV-1 and/or by aborting local infections. This principle has been demonstrated in macaques [49] and may over time provide an added benefit to using topical PrEP, i.e., an additional level of resistance through adaptive immunity. The nature of this immune response in microbicide-using women would be of major interest to the HIV vaccine field.

In this context, it is interesting to note that antibody responses in HIV-1 infected persons are dominated by IgG, whereas HIV-1-specific IgA appears to be impaired [25], [50], [51]. In contrast, exposed uninfected individuals develop nearly exclusively HIV-1-specific antibodies of the IgA type [16], as also seen in our study here. Furthermore, we have previously demonstrated that the initial antibody response to HIV-1 infection is directed to gp41 [23] and that the initial mucosal IgA response (in cervical lavage and seminal plasma) is also directed to gp41, with limited reactivity to other regions of the HIV-1 envelope [25]. In the exposed uninfected HPTN 035 women, however, six of the nine women with positive HIV-1-specific IgA in the vagina showed reactivity to gp120 and gp140, whereas only three reacted to gp41 epitopes. This finding is consistent with our work in another exposed uninfected cohort (CHAVI 002; United Kingdom and Uganda), where we detected anti-HIV-1 gp120 IgA, but not IgG, responses in blood (Shen *et al.,* unpublished). Thus, the results from both studies, with samples obtained from different geographic locations, converge on the same notion that HIV-1 gp120-specific IgA are induced in individuals that are (likely repeatedly) exposed to HIV-1, but are not part of the initial response to acute HIV-1 infection. Due to low numbers of women with mucosal HIV-1 specific IgA responses, we did not have the power to robustly determine if there was an association between sexual risk factors and the presence of vaginal HIV-1 IgA. The reasons for this skewing of genital HIV-1-specific antibody responses in exposed uninfected women toward the IgA subtype and toward HIV-1 gp120/gp140 remain unclear. However, these findings emphasize that further study of mucosal HIV-1-specific IgA responses in exposed uninfected individuals could uncover clues for purposefully driving a vaccine-induced response toward protective IgA-mediated mucosal immunity.

One caveat of the current study is that limited sample volume precluded testing of these mucosal antibodies for specific antiviral functions. Thus, we were not able to determine if the detected antibodies can block HIV-1 infection. Further studies comparing other methods for antibody elutions from wecks [52], [53] may provide improved antibody recovery. Other investigators have shown that monoclonal HIV-1 gp41-specific IgA antibodies derived from highly exposed seronegative individuals can neutralize HIV-1 and inhibit virion transcytosis through epithelial cells [40], [54], [55]. It is also encouraging that protection from simian/human immunodeficiency virus infection in macaques can in principle be achieved by passive administration of neutralizing and non-neutralizing HIV-1 inhibitory antibodies to the vagina [56], [57]. Thus, further studies are needed that collect larger volumes of mucosal fluids to continue investigations into the antiviral properties of mucosal IgA in HIV-1 exposed uninfected individuals, such as antibody-mediated movement arrest of HIV-1 in cervical mucus [58], virion capture [30], [59] and neutralization [11], [14], [16], [17].

Evaluation of mucosal immune responses is also crucial to interpret the findings of HIV-1 vaccine trials. The lack of such mucosal studies has left us with unanswered questions regarding the lack of efficacy of the HIV-1 vaccines tested in the Step, Phambili and HVTN 505 trials [60]–[63], as well as other potential correlates of protection conferred by the HIV-1 vaccine tested in the RV144 trial [64], [65]. These trials did not assess mucosal immune responses largely because of the lack of optimized and standardized procedures for mucosal specimen harvesting, including uncertainty over which specimens and sampling devices are best for a given research question. This is in part due to the variability of methods used to collect genital secretions, yielding different amounts of fluid and leading to sample dilution when using cervicovaginal lavage [19]–[21]. We compared three devices for harvesting undiluted vaginal secretions and found that Merocel sponges were preferred by clinicians and recovered similar amounts of total and HIV-1-specific antibodies as compared to Dacron and flocked nylon swabs. Thus, all three sampling devices could be used in future vaccine and topical PrEP trials where measurement of vaginal HIV-1-specific antibodies is desired.

Taken together, we report that genital HIV-1 envelope-specific antibodies in HIV exposed uninfected women in a large microbicide trial were predominantly of the IgA isotype. Strikingly, the epitope specificities of these Env-specific IgA were predominantly directed to gp120, unlike those IgAs induced in acute HIV-1 infection that predominantly target gp41 [23], [25]. Further study of these responses to determine their usefulness as markers of HIV-1 exposure as well as their protective potential is warranted.
